# Association of Epstein–Barr Virus (EBV) and Human Endogenous Retroviruses (HERV) with Multiple Sclerosis in Northwest of Iran

**DOI:** 10.1155/2023/8175628

**Published:** 2023-11-30

**Authors:** Sara Mafi, Dariush Savadi Oskoee, Hossein Bannazadeh Baghi, Arezou Azadi, Mahin Ahangar Oskouee

**Affiliations:** ^1^Neuroscience Research Center, Department of Neurology, Tabriz University of Medical Sciences, Tabriz, Iran; ^2^Clinical Research Development Unit, Booali Sina Hospital, Qazvin University of Medical Science, Qazvin, Iran; ^3^Infectious and Tropical Diseases Research Center, Tabriz University of Medical Sciences, Tabriz, Iran; ^4^Department of Microbiology and Virology, Tabriz University of Medical Sciences, Tabriz, Iran

## Abstract

**Materials and Methods:**

130 subjects were enrolled in a case-control study at two tertiary university hospitals from Tabriz (Imam and Razi), Iran. Of these, 65 subjects were MS patients serving as the case group, and 65 subjects were healthy individuals serving as the control group. After DNA extraction from all samples, the *EBER* region of EBV genome was used as the primer for the detection of EBV. RNA was extracted from PBMCs, and cDNA synthesis was performed by using Sina Gene kit. Subsequently, each sample was analysed by RT-PCR with two sets of primers to detect specifically multiple sclerosis retroviruses (MSRV) env, and RT-PCR was repeated for each HERV-W env. Positive sample was used in order to confirm the result.

**Results:**

In the case group, 19 (29.2%) patients were male and 46 (70.8%) patients were female. Nevertheless, in the control group, 21 (32.3%) subjects were male and 44 (67.7%) subjects were female. No significant difference was found between groups in gender (*p* = 0.70). The mean range in control and case groups was 33/38 ± 9/85 and 33.18 ± 8.65, respectively. No significant difference was found between groups in age (*p* = 0.902). 4 (6.2%) patients in case groups were found to be positive for EBV DNA (*p* = 0.119). Expression of the env gene of HERVs was observed in 10 (15.38%) and two (3.07%) specimens in the case and control groups (*p* = 0.030), separately. A comparison of the prevalence of the HERV ENV genome between the two study groups showed a significant difference (*p* = 0.005).

**Conclusion:**

The results of this study failed to show any difference between MS patients and healthy controls in the rate of EBV infection. It can be concluded that the expression of HERV-W/env genes may be involved in the development of MS in these patients.

## 1. Introduction

Multiple sclerosis (MS) is a chronic inflammatory disease of the central nervous system (CNS) that mainly affects young adults and causes inflammation and demyelination of the nerves, to varying degrees, leading to axonal injury and neuropathy. There is no known cure for multiple sclerosis, and treatments attempt to return function after an attack, prevent new attacks, and prevent disability [1]. Several genetic and environmental factors have been identified as critical risk factors for MS. The role of bacterial and viral agents in the initiation and progression of MS has been highlighted in several studies [2, 3]. Viruses such as Epstein–Barr virus (EBV) [4], HHV-6 [5], VZV [6], and HERV [7] have been studied in MS. Herpes viruses such as the EBV are thought to trigger the inflammatory cascade in the CNS which ultimately causes MS [8]. Accordingly, bacterial agents including *Chlamydia pneumonia*, among others, are also considered as potential contributing factors to the pathophysiology of MS [9–11]. However, the role of bacterial and viral pathogens as causative agents in MS is still a matter of debate. On one hand, it has been found that infection with EBV significantly increases the risk of MS development. On the other hand, the risk of MS is tremendously low in subjects who have not been infected with EBV [4]. Research has shown that increased levels of viral capsid antigen (VCA) IgG even in the maternal serum are linked to an elevated incidence of MS in the offspring [12]. Another study revealed that anti-EBNA nuclear antigen (EBNA) antibodies in the serum strongly predict the risk of MS and could be valuable in stratifying patients according to the MS risk score. [13].

Retroviruses have been linked to neurological diseases in humans and animals, such as tropical spastic paraparesis in humans with HTLV-1 [14]. This led to further study of the association of retroviruses in human MS. In 1989, Perron and colleagues first identified MS-related retroviral agents in the cerebrospinal fluid of MS patients, a member of the HERV-W family and known as MS (MSRV = multiple sclerosis retroviruses). MSRV have a complete proliferation cycle and are able to form extracellular infectious viruses [15]. The possible role of HERV in MS has long been considered. HERVs are derived from exogenous infectious retroviruses that integrated into genome germ line cells 70 to 30 million years ago and constitute approximately 8% of the human genome. During evolution, many HERV proviruses undergo extensive genetic modification, including deletion and mutation. They are present in the genome as defective copies unable to replicate, but some of them have a complete genome and are proliferative active [16]. HERV is composed of many families, which are multicopy. Each family comprises several loci in the human genome. The genome structure of HERVs has preserved the same genetic organization as exogenous retroviruses: four major viral genes: gag (encoding matrix and retroviral core), pol (reverse transcriptase and integrase), env (envelope) and pro (protease), and LTRs (long terminal repeat) regions at two different ends [17]. In various epidemiological investigations, a dependency between MS diagnosis and prognosis and MSRV/HERV-W has been demonstrated. Similarly, other HERVs have been investigated in relation to MS including HERV-H [18] and a HERV-K18 haplotype [19]. Perron et al. paid attention to HERV-W because MSRV (HERV-W) induces a T cell-mediated neuropathology in vivo [20]. Besides, MSRV/HERV-W envelope protein acts as a superantigen, causes polyclonal T lymphocyte activation, and stimulates a potent activation of innate immunity and the release of proinflammatory cytokines through toll-like receptor-4 (TLR-4) [20]. In addition, HERV-W env protein may instigate oligodendrocyte toxicity, inflammation-mediated [21]. HERVs can be activated by other viruses correlated with MS pathogenesis such as human herpesviruses-6 [22], Epstein–Barr (EBV) [22], and human herpesvirus-1 (HSV1), which produce immunopathology [23]. Our aim in the present study is to investigate the presence of mRNA env gene of HERV-W and EBV DNA in MS patients and healthy individuals in East Azerbaijan Province in Iran.

## 2. Materials and Methods

### 2.1. Study Design

This study was conducted as a case-control study performed at two tertiary university hospitals, Razi and Imam Reza, at Tabriz University of Medical Sciences, Tabriz, Iran, between 2015 and 2016. 130 subjects were enrolled to assess the association of infection with EBV, HERV-W, and MS. Of these, 65 subjects were MS patients serving as the case group, and 65 subjects were healthy individuals serving as the control group.

### 2.2. Inclusion and Exclusion Criteria

All patients diagnosed with MS and confirmed using comprehensive physical examinations, laboratory tests, and brain imaging, i.e., magnetic resonance imaging (MRI) performed by an experienced neurologist, were enrolled in this study. The control subjects were chosen from individuals who were considered normal, and evaluations had shown no evidence of MS or any other neurological disorders. Patients with other neurological disorders, those affected by pulmonary infections during the previous month, and those who received antiviral treatments were excluded from this study.

### 2.3. Study Methods

Fresh whole blood (5 ml) was taken in tubes containing anticoagulants from patients and controls. The samples were transported to the virology department of the Tabriz University of Medical Science. PBMCs were derived from blood by centrifugation at 15000 RPM in Ficol solution for 5 min, then immediately centrifugation was performed at 3000 RPM for 15 minutes. PBMCs were separated and kept at −80°C until used.

### 2.4. EBV DNA Extraction

**Figure 1:**
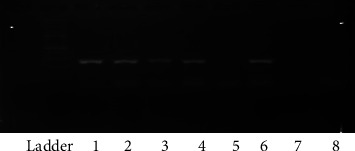
Agarose gel electrophoreses (1.5%) for *β*-globin gene. L = ladder (molecular weight 100 bp), 1–4, 6: positive samples (268 bp), c^−^: negative control, and 8: negative sample.

All DNA samples were extracted using the “Viral Nucleic Acid Extraction Kit I” (Yekta Tehiz Co., Taiwan), and based on its protocol, it was stored at −20°C until examination. Accordingly, the purity and integrity of the extracted DNA were validated via NanoDrop (NanoDrop, Epoch, USA) and PCR for *β*-globin gene, respectively (Figure 1). RNA was extracted from PBMCs with the Viral Nucleic Acid Extraction Kit (Favorgen, Cat No: fank001, Taiwan) following the manufacturer's instructions. RNA quality and concentration were assessed by using the NanoDrop spectrum photometer.

**Figure 2:**
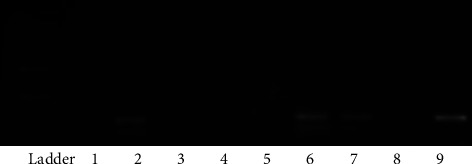
Agarose gel (1.5%) electrophoresis for EBV DNA. DNA ladder; molecular weight 100 bp; 1, 2, 6, and 7: positive MS samples for EBV DNA (each sample is duplicated), 3 and 4: negative samples, 8: negative control (sterile distilled water), 9: positive control (B95 cells) (108 bp).

The EBV EBER genome region was applied as the primer for uncovering EBV in all obtained samples (Table 1). DNA extracts from B95 cells and sterile distilled water were used as positive and negative controls, respectively. The following thermal cycling conditions were used: 69°C for 2′; followed by 38 cycles of 96°C for 2′, 58°C for 1′, 72°C for 2′; and with a final extension at 72°C for 10′. Visualization of amplified products was achieved using 1.5% agarose gel. PCR was repeated for each EBV-positive sample in order to confirm the result (Figure 2).

**Figure 3:**
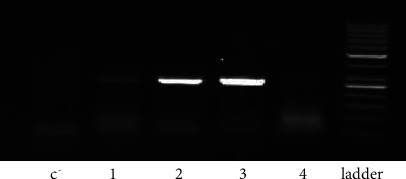
Agarose gel electrophoresis of the RT-PCR of HERV-W/env. C^−^: negative control, 1–3: HERV-W/env-positive samples (640 bp), 4: MSRV-negative sample, and L: 100 bp DNA ladder.

cDNA synthesis was performed by using the Sina Gene kit. Subsequently, each sample was analysed by RT-PCR with two sets of primers to detect, specifically, HERV-W env (Table 1). The following thermal cycling conditions were used: 64°C for 1′; followed by 35 cycles of 95°C for 1.30′, 60°C for 2′, 72°C for 2′; and with a final extension at 72°C for 10′. RT-PCR was repeated for each HERV-W env-positive sample in order to confirm the result, and RT-PCR was repeated for each HERV-W/env-positive sample in order to confirm the result (Figure 3).

### 2.5. Statistical Analysis

Statistical analyses were performed with SPSS software, version 20. Descriptive data were expressed using mean ± SD, numbers, and frequency. The relationship between infectious agents and MS was evaluated using a chi-square test. Additionally, logistic regression was used to adjust for the effect of confounding variables (if any). *p* < 0.05 was considered as statistically significant.

## 3. Results

A total of 130 patients (case and control) were enrolled in this study, of which 65 were in the case and 65 were in the control groups. In the case group, 19 (29.2%) patients were male and 46 (70.8%) patients were female. Nonetheless, in the control group, 21 (32.3%) subjects were male and 44 (67.7%) subjects were female. No significant difference was found between groups in gender (*p* = 0.70).  Table 2 represents the most common presenting signs and symptoms of the patients. In the patient's group, 4 (6.2%) patients were found to be positive for EBV DNA (Figure 1). The analysis found no meaningful difference between groups in this regard (*p* = 0.11). Likewise, no difference was found between male and female patients in EBV infection (*p* = 1.00).

RT-PCR assessment of samples from the isolated env region of HERV-W shows that the env region of virus was founded in 12/130 (9.2%), 10/65 (15/38%), and 2/65 (3/07%) in the case and control groups, respectively. (Figure 2). A comparison of the prevalence of the HERV ENV genome between the two study groups showed a significant difference (*p* = 0.005). Ratio of female to male was 3 : 1 (9 : 3) in patients who were viral positive. No statistically significant difference was observed regarding the gender distribution of the two groups with HERV (*p* = 0.587).

## 4. Discussion

Among infectious agents, EBV is a strong risk factor for the development of MS. It has been shown that the risk of MS is significantly lower in subjects who are seronegative for this virus. This so-called EBV theory shows that patients who had an episode of infection early in their lives would have a much higher risk of the development of MS in adulthood. By contrast, the risk of MS is much lower in peers who have escaped the infection at younger ages [27]. A recent comprehensive meta-analysis revealed that a history of EBV infection meaningfully escalates the risk of MS with a relative risk of 2.17 (95% CI 1.97–2.39) [28]. Sundqvist et al. confirmed the results of this meta-analysis with another study with an odds ratio of 1.89 (1.45–2.48 95% CI) [29]. These findings are not universal, and some remaining questions should be answered in this regard. Why do only some patients who are infected with EBV develop MS in later life? Is EBV a major causative agent in MS pathophysiology, is it just minor, or, as it has been asserted, is it just a red herring? [30]. Answers to these questions may also justify our results.

The results of our study could not recapitulate the findings mentioned earlier. Our investigation showed that only 4 out of 65 (6.2%) MS patients were positive for EBV DNA. Although in our sample, we demonstrated that patients with MS had a higher EBV infection (both current and previous) rate than their control counterparts, and the difference observed did not reach statistical significance.

Similarly, in a study by Mancuso et al. conducted on 51 patients with MS, cell-free or cell-associated viral DNA was evaluated in the cerebrospinal fluid (CSF) samples by real-time PCR. Moreover, viral loads were assessed in these samples. It was found that only one patient was positive for EBV DNA in cell-free CSF samples, and none was positive in cell-associated viral DNA samples. The authors, however, argued that lower rates of EBV DNA detection in the CSF could be due to the viral leakage to the CNS through cell trafficking from the periphery to the CSF and their removal from the CSF by the immune system in immunocompetent patients [31].

Cocuzza et al. tried to quantitatively assess EBV DNA in the CSF and blood samples of patients with relapsing-remitting MS. In the current study, EBV DNA was found to be present in 5.5% of cell-free and 18.2% of cell-associated CSF samples of MS patients. This was found to be 7.8% and 7.8% in controls, respectively. Plasma and peripheral blood mononuclear cells were positive for EBV DNA in 7.3% and 47.3% of the MS patients, whereas this was shown to be 5.8% and 31.4%, respectively, in the control group. The differences for all comparisons between the two groups were shown to be insignificant. This may imply the fact that no link exists between EBV and MS activity [32].

Al-Obaidi et al.'s study of EBV in multiple sclerosis found EBNA-1 antibody which is significantly higher in MS patients than in controls, especially at younger age groups (female), in early stages of the disease [33].

Buljevac et al., in the study of Epstein–Barr virus in MS, observed that chronic EBV reactivation may be associated with increased inflammatory activity as assessed by gadolinium-enhanced MRI lesions, which should be reproduced in a larger and independent dataset [34].

Biström et al., in 2020, in the study of the relationship between Epstein–Barr virus and MS, speculated that infection with EBV after adolescence increases the probability of MS [35].

Other hypotheses emerge from this evidence as follows: In light of that, it has been hypothesized that B cells that are activated by EBV turn against the nervous system, or rather the damage may result from the fact that the body tries to eliminate the virus at the cost of damage to the nervous system. This is commonly known as the bystander damage hypothesis [30]. However, evidence to support the bystander hypothesis is missing. There has been no study showing the presence of EBV-infected immune cells and EBV-directed CD8+ cells within the CNS. Nevertheless, a study by Serafini et al. would be able to show the activation of CD8+ T cells as representing evidence of cytotoxicity directed against plasma cells at regions of EBV-infected cells in the CNS [36]. The first hypothesis has also been subject to significant controversy, and pros [37] and cons [38] have either supported or rejected the hypothesis.

In our study, the existence of Env expression gene HERV-W/env in MS patients and control group has been qualitatively checked. Statistically significant differences marked that HERV env gene expression in MS patients (15/38%) is more than in controls (1.5%), and these results are consistent with other studies' results [39].

Arru et al., in 2007, by studying the association of endogenous retroviruses with multiple sclerosis and control subjects in different parts of Europe, observed a significant difference in the expression of HERV-W/MSRV pol and env genes between the patient and control groups [40].

Several studies have reported qualitative or semiquantitative differences between patients and controls for the presence or expression of MSRV/HERV-W [41–47].

In 1999, Lindeskog et al. [48], Brudekr et al. [18], Antong et al. in 2011 [10], and Gracia-Montojo et al. in 2013 [49] observed MSRV in studies of MS patients. Further correlation of HERV-W expression with MS: Sequential studies have demonstrated that HERV-W env protein was recognized in the serum sample of 73% of the MS patients [50], and also HERV-W/HERV-W env and pol RNAs were significantly expressed in peripheral blood mononuclear cells (PBMCs) and autopsied brain tissue from MS patients versus controls. Studies have shown that HERV-W envelope protein seems to act as a superantigen in MS patients and activate polyclonal T lymphocytes compared to the control group and stimulates a potent activation of innate immunity.

The release of proinflammatory cytokines through toll-like receptor-4 (TLR-4) and HERV-W env protein may instigate oligodendrocyte toxicity, inflammation-mediated [51]. In this regard, the current study provides information on the presence of the virus HERV-W/env in PBMCs from patients and control groups as a cofactor for MS. They are corroborated by an investigation of the expression of the env gene for this virus. Since they are so ubiquitous, genome detection alone does not necessarily mean viral activity. However, some of these studies did not report the presence of retroviruses in the studied MS patients [51, 52].

In addition, in the present study, we investigated the relationship between env gene expression of HERVs and sex, in which the difference was not statistically significant (*p* value = 0.649). This achievement contradicts the results of other studies that reported a close link between gender and env gene expression [47].

It cannot be neglected that our study had several shortcomings. First, the sample size of this study was limited. Therefore, studies with larger sample sizes are strongly recommended. We did not assess the presence of antibodies directed against the nucleus and capsid of EBV (anti-EBNA and anti-VCA antibodies, respectively) and/or detection of other HERV families such as H, K, or other HERV gene expression (pol, gag, and LTR).

If assessed, this might have resulted in more positive cases in our study. It has been shown that the risk of MS significantly decreases with migration from high to low prevalence regions, which is not consistent with EBV-causative theory [53, 54].

This signifies the fact that the role of EBV in MS may be modified by other environmental factors. Studies on the relationship between EBV and MS are ongoing around the world, but no consensus has yet been reached. As a result, it was decided to investigate the role of EBV in MS patients. Additionally, in gaining information about the previous incidence of infectious mononucleosis (IM) in our sample, we were largely dependent upon the self-report of the patients. Patients may confuse other mononucleosis-like syndromes with IM.

It seems that by gaining enough evidence to link viral and bacterial agents to MS, patients could benefit from appropriate prophylactic and therapeutic measures.

## 5. Conclusion

The results of this study failed to show any difference between MS patients and healthy controls in the rate of current or previous EBV infection. But even so from some results of the study, it can be concluded that the expression of HERV-env genes may be involved in the development of MS in these patients. However, to prove this finding, more extensive studies with a large sample size are needed. It is also recommended that people in the control group in this study, who were about 3.07% positive for the HERV, be followed up continuously to prevent the possible occurrence of MS, especially in people in high-risk ages. Due to the fact that these viruses belong to the family of retroviruses, by proving the connection between MS and the expression of these viruses, it is recommended that common antiretroviral drugs can be used to treat and prevent the progression of this disease. We highly recommend to study other regions of EBV genome and also study in other parts of Iran both EBV and HERV infection.

## Figures and Tables

**Table 1:** Primer sequence and size of the amplicon.

Target gene	Primer	Oligonucleotide sequence 5′-3′	Amplicon length	References	Annealing °C
Beta-globin	PCO4	AACTTCATCCACGTTCACC	250 bp	[24]	55
	GH20	GAAGAGCCAAGGACAGGTAC			

EBV: EBER	F	CCCTAGTGGTTTCGGACACACA	108 bp	[25]	58
	R	ACTTG CAAATGCTCTAGGCG			

HERV-W/env	F	TTCACTGCCCACACCCAT	640 bp	[26]	60
	R	GAGGTACCACAGACAAAAAATATTCCT			

**Table 2:** Demographic information of all patients and symptoms of patients with multiple sclerosis.

Patients information	*N* (135)%						
Sex	Case (65): *M* (19) = 29.2, *F* (46) = 70.8						
	Control (65): *M* (21) = 32.3, *F* (44) = 67.7						

Age (years)	Case (65): 15–66 (33.18 ± 8.65)						
	Control (65): 17–60 (33.38 ± 9.85)						

Level of education	Uneducated *N* (%)	Elementary *N* (%)	Middle school *N* (%)		High school *N* (%)	Bachelor's *N* (%)	University *N* (%)

Case (65)	3 (4.6)	4 (6.2)	5		35	19 (29.2)	0 (0.0)
Control (65)	3 (4.6)	5 (7.7)	4		34	10 (15.4)	8 (12.3)

Job	Employed *N* (%)			Unemployed *N* (%)			

Case (65)	62 (95.4)			3 (4.6)			
Control (65)	65 (100)			0 (0.0)			

Symptoms of MS patient	*N* (%)						

Upper body parts	3 (4.6)						
Lower body parts	6 (9.2)						
Unsteadiness	2 (3.1)						
Blurred vision	20 (30.8)						
Gait disturbance	1 (1.5)						
Paraparesis	6 (9.2)						
Paraplegia	20 (30.8)						
Incontinency	1 (1.5)						
Seizure	3 (4.6)						
Speech disorders	1 (1.5)						

History of MS	Case (65) (*N* %)			Control (65) (*N* %)			

Previous of infection family history of MS	2 (3.1)			0 (0.0)			
	1 (1.5)			0 (0.0)			

## Data Availability

The datasets that support the findings of this study are included within the article.

## References

[1] Compston A., Coles A. (2008). Multiple sclerosis. *The Lancet*.

[2] Mentis A. F. A., Dardiotis E., Grigoriadis N., Petinaki E., Hadjigeorgiou G. M. (2017). Viruses and multiple sclerosis: from mechanisms and pathways to translational research opportunities. *Molecular Neurobiology*.

[3] Cossu D., Yokoyama K., Hattori N. (2018). Bacteria-host interactions in multiple sclerosis. *Frontiers in Microbiology*.

[4] Levin L. I., Munger K. L., O’reilly E. J., Falk K. I., Ascherio A. (2010). Primary infection with the Epstein-Barr virus and risk of multiple sclerosis. *Annals of Neurology*.

[5] Leibovitch E. C., Jacobson S. (2014). Evidence linking HHV-6 with multiple sclerosis: an update. *Current opinion in virology*.

[6] Sotelo J., Ordoñez G., Pineda B., Flores J. (2014). The participation of varicella zoster virus in relapses of multiple sclerosis. *Clinical Neurology and Neurosurgery*.

[7] de la Hera B., Varadé J., García-Montojo M. (2014). Human endogenous retrovirus HERV-Fc1 association with multiple sclerosis susceptibility: a meta-analysis. *PLoS One*.

[8] Meier U. C., Cipian R. C., Karimi A., Ramasamy R., Middeldorp J. M. (2021). Cumulative roles for Epstein-Barr virus, human endogenous retroviruses, and human herpes virus-6 in driving an inflammatory cascade underlying MS pathogenesis. *Frontiers in Immunology*.

[9] Kakalacheva K., Münz C., Lünemann J. D. (2011). Viral triggers of multiple sclerosis. *Biochimica et Biophysica Acta (BBA)-Molecular Basis of Disease*.

[10] Antony J. M., DesLauriers A. M., Bhat R. K., Ellestad K. K., Power C. (2011). Human endogenous retroviruses and multiple sclerosis: innocent bystanders or disease determinants?. *Biochimica et Biophysica Acta (BBA)-Molecular Basis of Disease*.

[11] Libbey J. E., Cusick M. F., Fujinami R. S. (2014). Role of pathogens in multiple sclerosis. *International Reviews of Immunology*.

[12] Munger K., Zhang Z., Aivo J. (2017). *Maternal Levels of Epstein-Barr Virus IgG Antibodies and Risk of Multiple Sclerosis in Offspring in the Finnish Maternity Cohort (S44. 006)*.

[13] Munger K. L., Levin L. I., O’Reilly E. J., Falk K. I., Ascherio A. (2011). Anti-Epstein–Barr virus antibodies as serological markers of multiple sclerosis: a prospective study among United States military personnel. *Multiple Sclerosis Journal*.

[14] Grindstaff P., Gruener G. (2005). The peripheral nervous system complications of HTLV-1 myelopathy (HAM/TSP) syndromes. *Seminars in Neurology*.

[15] Oskari Virtanen J., Jacobson S., Jacobson S. (2012). Viruses and multiple sclerosis. *CNS and Neurological Disorders-Drug Targets*.

[16] Dolei A. (2006). Endogenous retroviruses and human disease. *Expert Review of Clinical Immunology*.

[17] Xu X., Zhao H., Gong Z., Han G. Z. (2018). Endogenous retroviruses of non-avian/mammalian vertebrates illuminate diversity and deep history of retroviruses. *PLoS Pathogens*.

[18] Brudek T., Christensen T., Petersen T., Møller-Larsen A. (2011). Expression of HERV-H/W env epitopes on PBMCs from MS patients with active disease. *Retrovirology*.

[19] de la Hera B., Varade J., García-Montojo M. (2013). Role of the human endogenous retrovirus HERV-K18 in autoimmune disease susceptibility: study in the Spanish population and meta-analysis. *PLoS One*.

[20] Perron H., Jouvin-Marche E., Michel M. (2001). Multiple sclerosis retrovirus particles and recombinant envelope trigger an abnormal immune response in vitro, by inducing polyclonal V*β*16 T-lymphocyte activation. *Virology*.

[21] Rolland A., Jouvin-Marche E., Viret C., Faure M., Perron H., Marche P. N. (2006). The envelope protein of a human endogenous retrovirus-W family activates innate immunity through CD14/TLR4 and promotes Th1-like responses. *The Journal of Immunology*.

[22] Christensen T. (2006). The role of EBV in MS pathogenesis. *International MS Journal*.

[23] Ruprecht K., Obojes K., Wengel V. (2006). Regulation of human endogenous retrovirus W protein expression by herpes simplex virus type 1: implications for multiple sclerosis. *Journal of NeuroVirology*.

[24] Ahangar Oskouee M., Shahmahmoodi S., Jalilvand S. (2014). No evidence of mammary tumor virus env gene-like sequences among Iranian women with breast cancer. *Intervirology*.

[25] Kadivar M., Monabati A., Joulaee A., Hosseini N. (2011). Epstein-Barr virus and breast cancer: lack of evidence for an association in Iranian women. *Pathology and Oncology Research*.

[26] Laufer G., Mayer J., Mueller B. F., Mueller-Lantzschand N., Ruprecht K. (2009). Analysis of transcribed human endogenous retrovirus W env loci clarifies the origin of multiple sclerosis-associated retrovirus envsequences. *Retrovirology*.

[27] Warner H., Carp R. (1981). Multiple sclerosis and Epstein-Barr virus. *Multiple sclerosis and Epstein-Barr virus*.

[28] Handel A. E., Williamson A. J., Disanto G., Handunnetthi L., Giovannoni G., Ramagopalan S. V. (2010). An updated meta-analysis of risk of multiple sclerosis following infectious mononucleosis. *PLoS One*.

[29] Sundqvist E., Sundström P., Lindén M. (2012). Epstein-Barr virus and multiple sclerosis: interaction with HLA. *Genes and Immunity*.

[30] Burnard S., Lechner-Scott J., Scott R. J. (2017). EBV and MS: major cause, minor contribution or red-herring?. *Multiple sclerosis and related disorders*.

[31] Mancuso R., Hernis A., Cavarretta R. (2010). Detection of viral DNA sequences in the cerebrospinal fluid of patients with multiple sclerosis. *Journal of Medical Virology*.

[32] Cocuzza C. E., Piazza F., Musumeci R. (2014). Quantitative detection of Epstein-Barr virus DNA in cerebrospinal fluid and blood samples of patients with relapsing-remitting multiple sclerosis. *PLoS One*.

[33] Al-Obaidi A. B., Ali Z. A., Rasool Almashta S. A., Ghazi H. F. (2022). The potential role of Epstein-Barr virus in Multiple sclerosis molecular and serological study. *Wiadomosci Lekarskie*.

[34] Buljevac D., Van Doornum G. J. J., Flach H. Z. (2005). Epstein-Barr virus and disease activity in multiple sclerosis. *Journal of Neurology, Neurosurgery and Psychiatry*.

[35] Biström M., Jons D., Engdahl E. (2021). Epstein–Barr virus infection after adolescence and human herpesvirus 6A as risk factors for multiple sclerosis. *European Journal of Neurology*.

[36] Serafini B., Rosicarelli B., Franciotta D. (2007). Dysregulated Epstein-Barr virus infection in the multiple sclerosis brain. *Journal of Experimental Medicine*.

[37] Sorensen P. S., Blinkenberg M. (2016). The potential role for ocrelizumab in the treatment of multiple sclerosis: current evidence and future prospects. *Therapeutic advances in neurological disorders*.

[38] Pender M. P., Csurhes P. A., Smith C. (2014). Epstein–Barr virus-specific adoptive immunotherapy for progressive multiple sclerosis. *Multiple Sclerosis Journal*.

[39] Morandi E., Tanasescu R., Tarlinton R. E. (2017). The association between human endogenous retroviruses and multiple sclerosis: a systematic review and meta-analysis. *PLoS One*.

[40] Mostafa A., Jalilvand S., Shoja Z. (2017). Multiple sclerosis-associated retrovirus, Epstein-Barr virus, and vitamin D status in patients with relapsing remitting multiple sclerosis. *Journal of Medical Virology*.

[41] Mameli G., Astone V., Arru G. (2007). Brains and peripheral blood mononuclear cells of multiple sclerosis (MS) patients hyperexpress MS-associated retrovirus/HERV-W endogenous retrovirus, but not Human herpesvirus 6. *Journal of General Virology*.

[42] Dolei A., Serra C., Mameli G. (2002). Multiple sclerosis–associated retrovirus (MSRV) in Sardinian MS patients. *Neurology*.

[43] Garson J. A., Tuke P. W., Giraud P., Paranhos-Baccala G., Perron H. (1998). Detection of virion-associated MSRV-RNA in serum of patients with multiple sclerosis. *The Lancet*.

[44] Komurian-Pradel F., Paranhos-Baccala G., Bedin F. (1999). Molecular cloning and characterization of MSRV-related sequences associated with retrovirus-like particles. *Virology*.

[45] Perron H., Geny C., Laurent A. (1989). Leptomeningeal cell line from multiple sclerosis with reverse transcriptase activity and viral particles. *Research in Virology*.

[46] Yi J. M., Kim H. M., Kim H. S. (2004). Expression of the human endogenous retrovirus HERV-W family in various human tissues and cancer cells. *Journal of General Virology*.

[47] Montojo M. G., Hera B. D. L., Varadé J., Encarnación A. D. L., Camacho I. (2014). HERV-W polymorphism in chromosome X is associated with multiple sclerosis risk and with differential expression of MSRV. *Retrovirology*.

[48] Lindeskog M., Mager D. L., Blomberg J. (1999). Isolation of a human endogenous retroviral HERV-H element with an open env reading frame. *Virology*.

[49] Garcia-Montojo M., Dominguez-Mozo M., Arias-Leal A. (2013). The DNA copy number of human endogenous retrovirus-W (MSRV-type) is increased in multiple sclerosis patients and is influenced by gender and disease severity. *PLoS One*.

[50] Perron H., Germi R., Bernard C. (2012). Human endogenous retrovirus type W envelope expression in blood and brain cells provides new insights into multiple sclerosis disease. *Multiple sclerosis journal*.

[51] de Villiers J. N. P., Treurnicht F. K., Warnich L., Carr J., van Rensburg S. J., Kotze M. J. (2006). Analysis of viral and genetic factors in South African patients with multiple sclerosis. *Metabolic Brain Disease*.

[52] Ruprecht K., Gronen F., Sauter M., Best B., Rieckmann P., Mueller-Lantzsch N. (2008). Lack of immune responses against multiple sclerosis—associated retrovirus/human endogenous retrovirus W in patients with multiple sclerosis. *Journal of NeuroVirology*.

[53] Ascherio A., Munger K. (2010). 99th Dahlem conference on infection, inflammation and chronic inflammatory disorders: Epstein–Barr virus and multiple sclerosis: epidemiological evidence. *Clinical and Experimental Immunology*.

[54] Ascherio A., Munger K. L. (2007). Environmental risk factors for multiple sclerosis. Part II: noninfectious factors. *Annals of Neurology*.

